# Is Accurate Lumen Segmentation More Important than Outlet Boundary Condition in Image-Based Blood Flow Simulations for Intracranial Aneurysms?

**DOI:** 10.1007/s13239-023-00675-1

**Published:** 2023-08-15

**Authors:** Jana Korte, Samuel Voß, Gábor Janiga, Oliver Beuing, Daniel Behme, Sylvia Saalfeld, Philipp Berg

**Affiliations:** 1 Forschungscampus STIMULATE, University of Magdeburg, Magdeburg, Germany; 2 Department of Fluid Dynamics and Technical Flows, University of Magdeburg, Magdeburg, Germany; 3 Department of Radiology, AMEOS Hospital, Bernburg, Germany; 4 Department of Neuroradiology, University Hospital of Magdeburg, Magdeburg, Germany; 5 Department of Computer Science and Automation, Ilmenau University of Technology, Ilmenau , Germany; 6 Department of Medical Engineering, University of Magdeburg, Magdeburg, Germany

**Keywords:** Intracranial aneurysm, Outlet boundary condition, Hemodynamic parameters, Lumen segmentation, Flow-splitting

## Abstract

**Purpose:**

Image-based blood flow simulations are increasingly used to investigate the hemodynamics in intracranial aneurysms (IAs). However, a strong variability in segmentation approaches as well as the absence of individualized boundary conditions (BCs) influence the quality of these simulation results leading to imprecision and decreased reliability. This study aims to analyze these influences on relevant hemodynamic parameters within IAs.

**Methods:**

As a follow-up study of an international multiple aneurysms challenge, the segmentation results of five IAs differing in size and location were investigated. Specifically, five possible outlet BCs were considered in each of the IAs. These are comprised of the zero-pressure condition (BC1), a flow distribution based on Murray’s law with the exponents n = 2 (BC2) and n = 3 (BC3) as well as two advanced flow-splitting models considering the real vessels by including circular cross sections (BC4) or anatomical cross sections (BC5), respectively. In total, 120 time-dependent blood flow simulations were analyzed qualitatively and quantitatively, focusing on five representative intra-aneurysmal flow and five shear parameters such as vorticity and wall shear stress.

**Results:**

The outlet BC variation revealed substantial differences. Higher shear stresses (up to Δ9.69 Pa), intrasaccular velocities (up to Δ0.15 m/s) and vorticities (up to Δ629.22 1/s) were detected when advanced flow-splitting was applied compared to the widely used zero-pressure BC. The tendency of outlets BCs to over- or underestimate hemodynamic parameters is consistent across different segmentations of a single aneurysm model. Segmentation-induced variability reaches Δ19.58 Pa, Δ0.42 m/s and Δ957.27 1/s, respectively. Excluding low fidelity segmentations, however, (a) reduces the deviation drastically (>43%) and (b) leads to a lower impact of the outlet BC on hemodynamic predictions.

**Conclusion:**

With a more realistic lumen segmentation, the influence of the BC on the resulting hemodynamics is decreased. A realistic lumen segmentation can be ensured, e.g., by using high-resolved 2D images. Furthermore, the selection of an advanced outflow-splitting model is advised and the use of a zero-pressure BC and BC based on Murray’s law with exponent n = 3 should be avoided.

## Introduction

Image-based hemodynamic simulations enable the acquisition of patient-specific blood flow information at high spatial and temporal resolutions. This is particularly helpful in assessing the individual state of vascular diseases and evaluating the progression and associated risk quantitatively. One prominent example of a serious neurovascular pathology is represented by an intracranial aneurysm (IA). In IAs, permanent dilatation of the cerebral arterial vessel wall might cause neurological symptoms due to space-occupying effects or can lead to a subarachnoid hemorrhage associated with high rates of mortality and morbidity [1]. IAs can present in different sizes, shapes, locations and even as multiple IAs. The processes leading to aneurysm development are poorly understood, however once an IA occurs, various hemodynamic metrics are among the most relevant factors associated with aneurysm rupture [2, 3]. Therefore, improvements in the understanding of patient-specific hemodynamic environments are mandatory concerning the development and on top of that the criteria for the treatment of IAs [1].

Cerebrovascular simulations of IAs claim to provide the desired individual flow patterns, nevertheless the vascular geometry is often the only true patient-specific aspect. Several assumptions and simplifications are necessary for these flow simulations. Within the last decade, various studies focused on these individual aspects related to hemodynamic simulations in IAs. Exemplarily, Voß et al. [4] compared fluid–structure-interaction simulations based on patient-specific and constant vessel wall thicknesses and highlighted the need for realistic data acquisition. Apart from the vessel wall, simulations based on computational fluid dynamics (CFD) require sufficient inlet and outlet boundary conditions (BCs) [5, 6]. Valen-Sendstad et al. [7] investigated the scaling of inflow rates according to the vessel diameter and demonstrated good agreement between the square law and physiological flow rates. The common zero-pressure outlet BC is widely used in cerebral flow simulation studies [8–10]. The importance of sufficient outflow BCs is emphasized by Chnafa et al. [11], who introduced an advanced flow-splitting tool for an arbitrary vessel geometry. Saalfeld et al. [12] further developed this flow-splitting approach by additionally considering the vessel morphology. Consequently, comparing all five methods within the tool by Saalfeld et al., the most realistic aspects are taken into account. The overall implementation is still lacking in state-of-the-art research and since the investigation of the vascular region of interest, including a rising number of outflow cross sections, continuously becomes more common, the consideration of realistic outflow BCs becomes more and more important.

Therefore, this study focuses on emphasizing the importance of outlet BCs for IA simulations by applying advanced splitting methods and the related comparison between widely used (e.g., zero pressure) and state-of-art (flow-splitting) outflow BCs. This comparison is conducted as an extension of the Multiple Aneurysms AnaTomy CHallenge 2018 (MATCH) [13] in which 26 groups from 13 countries contributed the segmentation results of five patient-specific IAs. The consideration of different segmentations is important to include the entirety of manual processing, since the local vessel cross-sections interact with the flow-splitting. Hence, a real-world variability of possible segmentations is considered, allowing for a realistic assessment of outflow strategy related effects. Finally, it enables the estimation of whether the influence of the outlet BC dominates over the segmentation.

## Methods

### Patient Data

In the previous MATCH studies focusing on the impact of segmentation variability, five IAs were analyzed, all found in a patient with a subarachnoid hemorrhage [13, 14]. As described in Berg et al. [13] two IAs (A and B) were located at the right middle cerebral artery (vessel model 1), two (C and D) at the left middle cerebral artery (vessel model 2) and one (E) at the left posterior inferior cerebellar artery (vessel model 3). All three vessel models are part of this study’s analysis.

### Medical Imaging and Segmentation

To obtain the geometric information of the patient-specific IA, 3D rotational angiography (0.28 mm isotropic spatial resolution) was performed on an Artis Q angiography system (Siemens Healthineers AG, Forchheim, Germany). Afterwards, segmentation was carried out by 26 groups in the framework of the MATCH study [13]. To assure a wide range of segmentation approaches, no instructions were given. Further details concerning this challenge can be found in [13]. For two of the IAs, highly resolved 2D reference images were available, enabling the quantification of the segmentation results.

**Fig. 1:**
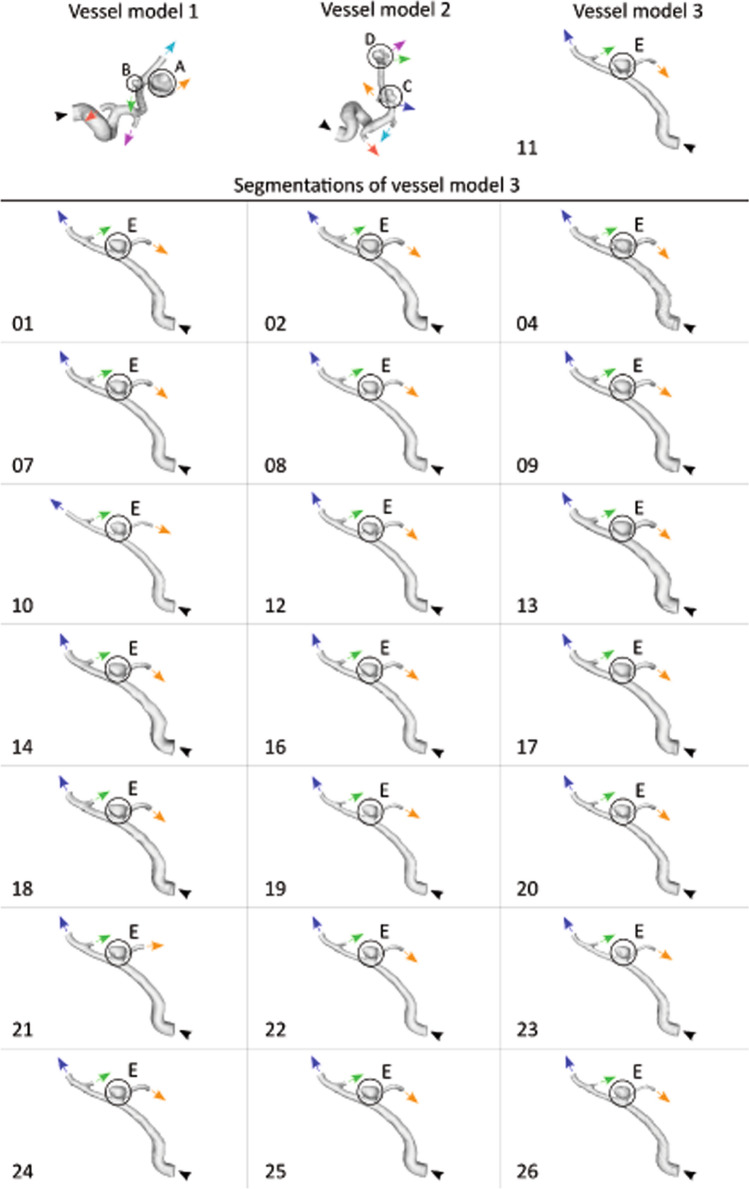
Illustration of the 3 vessel models and the 22 segmentations of vessel model 3 comprising aneurysm E. Each outlet is marked with an arrow. Outlet extension is not presented here. Inlets are marked with a black arrowhead. Aneurysms are highlighted with a circle. Outlet order of vessel model 3 (O1: yellow arrow, O2: green arrow, O3: blue arrow) is from right to left. Segmentation results of vessel model 3 of group 3, 5, 6 and 15 were discarded. Segmentation result of group 11 of vessel model 3 is shown in the first row

One of the two aneurysms used for validation shows high segmentation variability over all groups compared to the reference 2D DSA data [14–16]. Variations in the segmentation are of high interest when analyzing the segmentations’ influence, hence, this IA (aneurysm E) was chosen to be analyzed containing every group’s segmentation result in this study. Figure 1 illustrates the three vessel models, including the five aneurysms and the final underlying segmentations of vessel model 3 (comprising IA E). The segmentations of four groups could not be used due to strong artifacts (3 and 5) [14] and a missing second outlet (6 and 15), respectively. Concerning the segmentations of IA E, clear differences in smoothing (e.g., groups 9 and 17 compared to groups 4 and 26) and in neck size, with a bigger neck (groups 13 and 17) and smaller neck (groups 8 and 10) can be seen. The aneurysm appears to show elevations and uneven spots (e.g., 7 and 23). This applies as well to the vessel as shown for groups 1, 2, 11, 13, 14 and 18.

Note the individual outlet cross-sections of vessel model 1 (O1: red arrow, O2: purple arrow, O3: green arrow, O4: yellow arrow, O5: blue arrow), vessel model 2 (O1: red arrow, O2: light-blue arrow, O3: dark-blue arrow, O4: orange arrow, O5: green arrow, O5: purple arrow) and vessel model 3 (O1: yellow arrow, O2: green arrow, O3: blue arrow), where the different outflow BCs are applied (see "Outlet Boundary Conditions" and "Hemodynamic Simulations" sections).

### Outlet Boundary Conditions

In total, five outlet BCs were chosen. First, the most commonly applied zero-pressure condition (BC1) with a constant pressure *p* = 0 at each outlet was used. Second, two flow-splitting techniques inspired by Murray’s law were considered. This law is the particular case of the common proportional relation between vessel flow rate and vessel diameter [11]. Taking the mass conservation into account and leaving out the branch junctions of the vessel, this law is raised to two power exponents n = 2 (BC2) and n = 3 (BC3) [11, 17, 18]. Equation (1) contains the Murray’s law relationship. The flow rates ${Q}_{i}$ and diameters ${D}_{i}$ at each vessel cross section (i) are proportional.

1 $$\frac{{Q}_{i}}{\sum {Q}_{i}}={\left(\frac{{D}_{i}}{\sum {D}_{i}}\right)}^{n}.$$

Next, a flow-splitting technique adapted from Eq. (1) applied locally at each bifurcation, and based on the circular vessel cross sections (BC4) [11], is implemented. Here, the power exponent is set to n = 2. This BC4 is closely related to BC2, since it is using the equal law and relationship. Nevertheless, the improvement is the local investigation of the flow splitting at each branch junction [11].

2 $$\frac{{Q}_{1}}{{Q}_{2}}={\left(\frac{{D}_{1}}{{D}_{2}}\right)}^{2}.$$

Finally, the fifth BC represents an advanced flow-splitting model, which takes the true local vessel cross-section into account (BC5) [12]. The approach used for BC4 is adapted by replacing the squared diameter with the real luminal cross-sectional area. This area is approximated as a polygonal surface based on a raytracing approach, with rays originating at the vessel centerline and pointing at the surface border [12].

3 $$\frac{{Q}_{1}}{{Q}_{2}}=\frac{{A}_{1}}{{A}_{2}}.$$

Since the latter method considers the most variance in the geometry, it serves as the reference model in the overall comparison. Due to missing in vivo flow data, it is applied as a substitute for a ground truth. Equation-based, BCs 2, 4 and 5 show relations in accessing the splitting value, each containing an improvement to the previous one (from 2 to 4 and to 5). BC1 and 3 fall out of this comparable basis, since they refer only to a constant pressure or a relationship with exponent = 3 (BC3), whereas the latter cannot be related to the vessel cross-section.

### Hemodynamic Simulations

As indicated in "Medical Imaging and Segmentation" section, the segmentation results of 22 MATCH participants were considered in this study. To ensure consistent conditions for all hemodynamic simulations, all models were spatially discretized using identical mesh settings (mesh base size of Δx = 0.07–0.09 mm; cell count of 1.9–2.8 million cells). For further information on the patient selection, segmentation and meshing, see Berg et al. [13] and Voß et al. [14].

Inlet BCs were set to a representative time-varying flow rate based on [19] and adapted depending on the individual inlet cross section [13, 20]. To achieve a fully developed flow profile, the inlet and outlet cross-sections were virtually extruded in the normal direction [21] by at least six times the nominal diameter. This chosen time varying inflow leads to transient outlet flow curves. The flow was assumed to be laminar, and a constant time step size of Δt = 0.001 s and a convergence criterion (continuity) of 1E−04 were set. Since the application of blood as a Newtonian or non-Newtonian fluid in hemodynamic simulations of IAs is verifiably insignificant, blood was considered Newtonian (viscosity μ = 0.004 Pa s) [22, 23]. Blood density was assumed to be incompressible with ρ = 1055 kg/m^3^.

In total, 120 time-dependent blood flow simulations were carried out using STAR CCM+ 12.02 (Siemens Product Lifecycle Management Software, Inc., Plano, TX, USA) considering five different outflow BCs for vessel model 1 containing aneurysms A and B, vessel model 2 containing aneurysms C and D and 22 segmentations of vessel model 3 containing aneurysm E (recall "Outlet Boundary Conditions" section). Each hemodynamic simulation contained three cardiac cycles; however, the first two cycles were discarded and only the third one was analyzed.

### Analysis

**Fig. 2:**
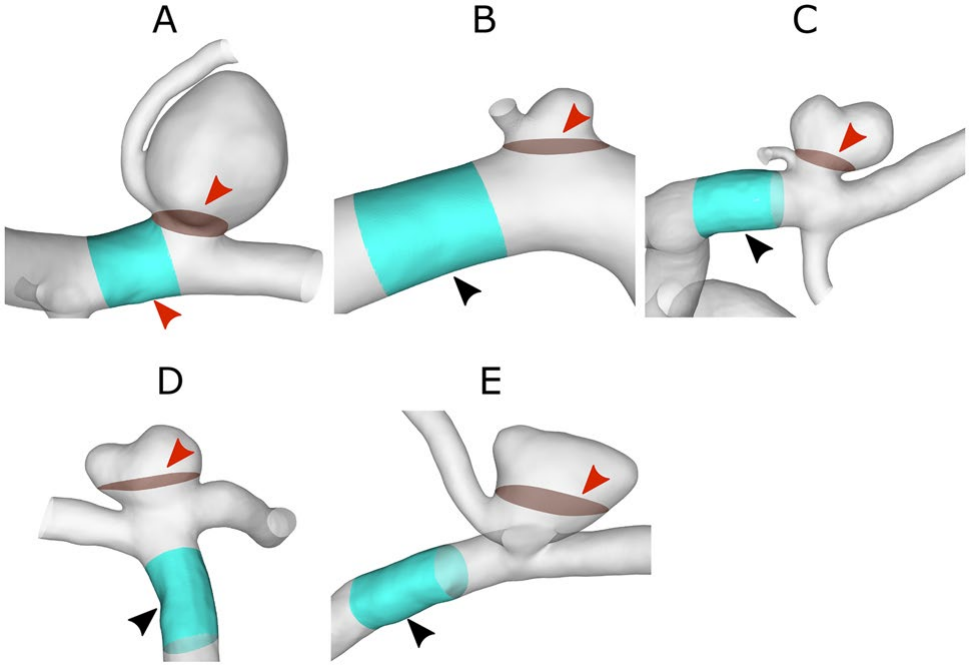
Placement of the parent vessel part (colored in cyan) and the ostium surface area inside the aneurysm (colored red and marked with a red arrowhead) for all IAs (A–E)

Relevant hemodynamic parameters were analyzed to evaluate the dependencies of the five different outflow BCs on the numerical flow predictions. The analysis was carried out qualitatively as well as quantitatively. For the calculation of hemodynamic parameters and analysis of the results ANSYS EnSight 2021 v10.2 (ANSYS, Inc., Canonsburg, PA, USA) and MATLAB R2020b (MathWorks, Inc., Natick, MA, USA) were used. Specific quantities, which will be described in "Flow Parameters" and "Shear Parameters" sections, require the definition of a parent vessel section for reference. This parent vessel was chosen to be a part of the vessel proximal to the aneurysm neck. Figure 2 shows the selected parent vessel part for each aneurysm as well as the selected ostium plane, which is the narrowest common surface area at the neck of the aneurysm over all segmentations [5, 14].

#### Flow Parameters

The blood velocity (V) served as the basis for the following calculations. The temporal mean over one cardiac cycle was taken for all quantifications. Aneurysm-related parameters were spatially averaged. The flow rates (Q) were calculated at all outlet surfaces of the overall geometry (outlet flow), respectively.

Vorticity (ω) describes the self-rotation of each fluid element around its own axis. To calculate ω, the velocity components (u, v, w) of the velocity directions (x, y, z) are taken into account [24].

ω components are designated as ${{\varvec{\zeta}}}_{x}$, ${{\varvec{\zeta}}}_{y}$, ${{\varvec{\zeta}}}_{z}$ with u, v, w—velocity components of velocity directions x, y, z:

4 $${{\varvec{\zeta}}}_{x}=\frac{{\partial }_{w}}{{\partial }_{y}}-\frac{{\partial }_{v}}{{\partial }_{z}},$$

5 $${{\varvec{\zeta}}}_{y}=\frac{{\partial }_{u}}{{\partial }_{z}}-\frac{{\partial }_{w}}{{\partial }_{x}},$$

6 $${{\varvec{\zeta}}}_{z}=\frac{{\partial }_{v}}{{\partial }_{x}}-\frac{{\partial }_{u}}{{\partial }_{y}}.$$

The kinetic energy (KE) describes the part of the energy related to motion inside the volume part of the IA [25]. The kinetic energy ratio (KER) describes the ratio between KE occurring inside the aneurysm in relation to the parent vessel [21, 26, 27].

7 $$KE= \frac{1}{2}*\rho {V}^{2},$$

8 $$KER= \frac{{KE}_{Aneurysm}}{{KE}_{parentvessel}}.$$

The oscillatory velocity index (OVI) characterizes the impact of vorticity and gives information about the temporal velocity fluctuation. Hence, the vortex formation inside a flow field can be examined. OVI ranges between 0 (steady flow) and 0.5 (high temporal changes) [28–30] and was calculated as follows:

9 $$OVI= \frac{1}{2} * \left\{ 1 - \frac{ \frac{1}{T }\left| {\int }_{0}^{T}V dt \right|}{\frac{1}{T} {\int }_{0}^{T}\left| V \right|dt } \right\} \quad 0<OVI<0.5.$$

#### Shear Parameters

Concerning the shear parameters occurring at the aneurysm sac, the time-averaged wall shear stress (TAWSS) and low shear area (LSA) were calculated [14, 25, 31].

10 $$TAWSS= \frac{1}{T}{\int }_{0}^{T}\left|WSS\right|dt.$$

LSA is the aneurysm wall area which is exposed to TAWSS values lower than the TAWSS at the parent vessel wall minus its standard deviation [18, 27]. This area is then normalized by the aneurysm surface area.

11 $$TAWSS{ }_{boundary}= TAWS{S}_{parentvessel}-SD\left(TAWS{S}_{parentvessel}\right),$$

12 $$LSA= \frac{Are{a}_{aneurysm(TAWSS<TAWSS{ }_{boundary})}}{Are{a}_{aneurysm}}.$$

**Fig. 3:**
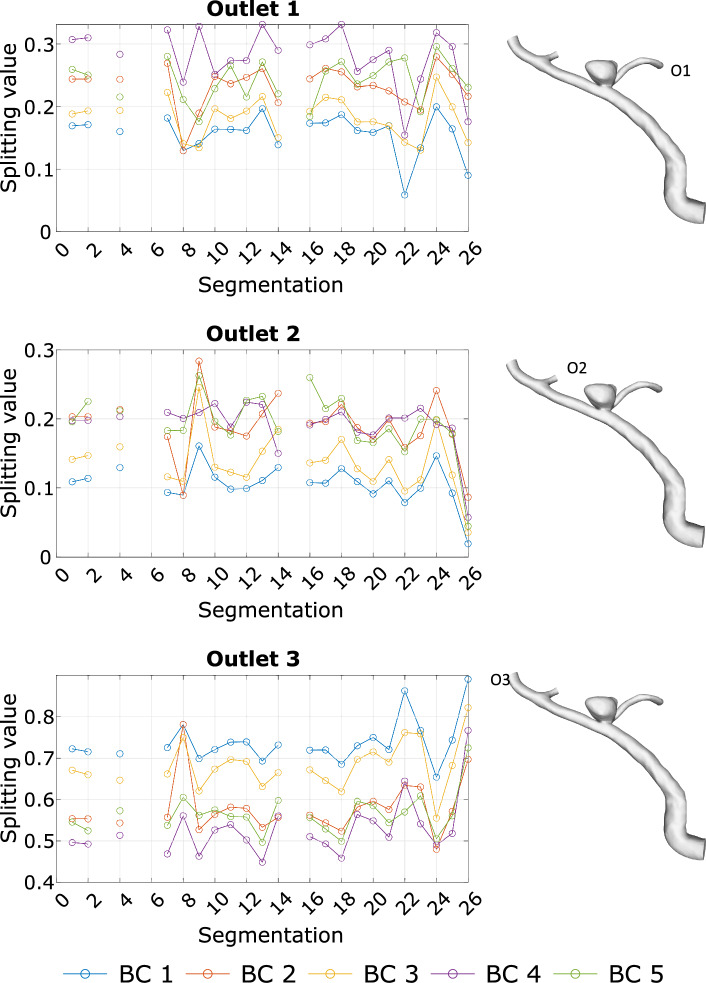
Overview of the different flow-splitting values for each segmentation of vessel model 3 for each outlet and BC. BC1 (blue): p0, BC2 (red): Murray n = 2, BC3 (yellow): Murray n = 3, BC4 (purple): Chnafa et al. [11], BC5 (green): Saalfeld et al. [12]. The location of the corresponding outlet is labeled on the right

Finally, to enable a relative comparison of each aneurysm, the TAWSS was normalized by using the ratio between the shear stress on the aneurysm wall and the parent vessel wall (nAWSS) [31] (recall Fig. 3).

13 $$nTAWSS= \frac{{TAWSS}_{aneurysm}}{{TAWSS}_{parent vessel}}.$$

Mean values describe the spatial mean inside the IA sac and on the IA sac surface.

## Results

Overall, 120 hemodynamic simulations were carried out in order to analyze the influence of the different BCs and segmentations on the flow and shear parameters in IAs. First, the resultant flow-splitting values are presented. Second, flow visualizations and qualitative wall parameters are shown. Third, the interplay between segmentation and outlet BC is evaluated.

### Splitting Values

In Table 1 the outlet splitting values for each BC are listed for the vessel models 1–3. Each outlet BC leads indirectly (BC1) or directly (BC2–5) to a certain ratio of the vessel outflow rate. When applying BC1, the splitting values are calculated after each hemodynamic simulation based on the corresponding outflow. For all other BCs, the splitting values are calculated based on the geometric model prior to each simulation. The splitting value signifies its fraction of the overall outflow rate.

**Table 1:** Resultant splitting values for the outlets of each vessel model 1–3 (rows) and each BC1–5 (columns) and the deviation of BC1–4 to BC5 in %

		BC1	BC2	BC3	BC4	BC5
Vessel model 1	Outlet 1	0.513 (+41)	0.401 (+25)	0.465 (+35)	0.345 (+13)	0.301
	Outlet 2	0.209 (−9)	0.207 (−10)	0.173 (−31)	0.239 (+5)	0.227
	Outlet 3	0.002 (−900)	0.016 (−25)	0.004 (−400)	0.019 (−5)	0.020
	Outlet 4	0.011 (−555)	0.054 (−33)	0.023 (−213)	0.069 (−4)	0.072
	Outlet 5	0.265 (−44)	0.322 (−18)	0.335 (−14)	0.033 (−1055)	0.381
Vessel model 2	Outlet 1	0.397 (+36)	0.295 (+14)	0.351 (+28)	0.255 (+0)	0.254
	Outlet 2	0.187 (−1)	0.143 (−31)	0.119 (−58)	0.218 (+14)	0.188
	Outlet 3	0.011 (−1009)	0.030 (−307)	0.011 (−1009)	0.139 (+12)	0.122
	Outlet 4	0.086 (+59)	0.095 (+63)	0.064 (+45)	0.026 (−35)	0.035
	Outlet 5	0.138 (−56)	0.181 (−19)	0.170 (−26)	0.194 (−11)	0.215
	Outlet 6	0.180 (−3)	0.256 (+27)	0.284 (+35)	0.167 (−11)	0.186
Vessel model 3	Outlet 1	0.164 (−62)	0.236 (−13)	0.181 (−47)	0.274 (+3)	0.266
	Outlet 2	0.098 (−82)	0.182 (+2)	0.123 (−45)	0.190 (+6)	0.178
	Outlet 3	0.738 (+25)	0.581 (+4)	0.697 (+20)	0.536 (−4)	0.555

Figure 3 presents the splitting values for vessel model 3 for each outlet BC over the different segmentations. As indicated in Fig. 1, outlet O3 is the main outlet of vessel model 3 with the highest splitting values. An equal curve of the BCs over the different segmentations is observed.

### Outlet Boundary Condition

**Fig. 4:**
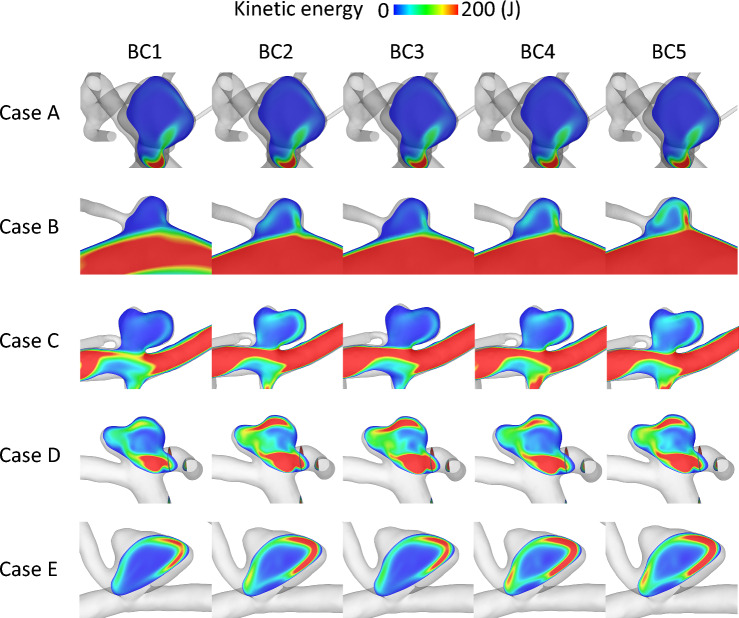
Contours through aneurysm model colored with KE within each IA (rows) for each BC1–5 (columns)

To compare the impact of the BC on the aneurysmal flow, a plane through the aneurysm colored according to the temporal mean KE is shown in Fig. 4 for all IAs and BCs. Comparing the aneurysms column-wise illustrates the effect of the BC, and the different IA models are presented row-wise, respectively. Regardless of the BC, the incoming flow forms a vortex inside the aneurysm with higher KE closer to the aneurysm wall and lower KE inside the sac for each IA. The difference between the impact of the BCs is clearly visible within the magnitude of KE. With BC1 and 3, the KE inside the aneurysm tends to be smaller than with BC2, 4 and 5. The highest values are shown within BC4 and 5. This is consistent for all 5 IAs.

**Fig. 5:**
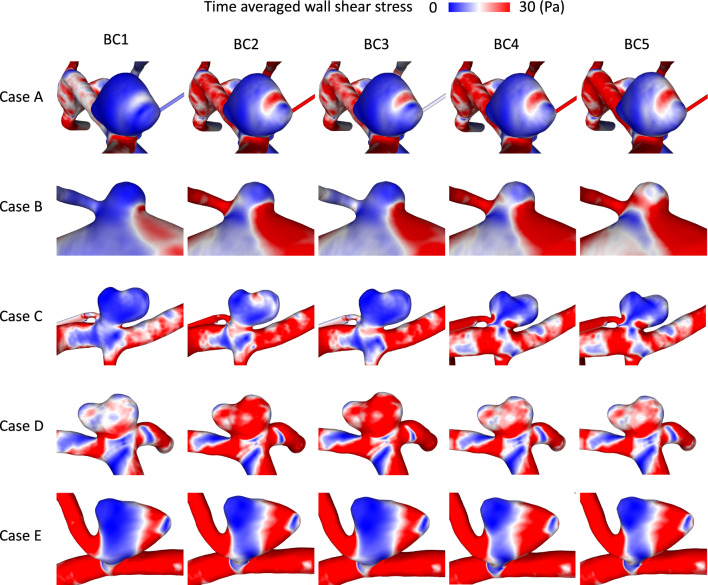
Illustration of the TAWSS occurring on the luminal surface of each IA (rows) considering BC1–5 (columns)

Figure 5 shows the TAWSS over one cardiac cycle for all IAs and BCs. The wall parameter shows an equal pattern for each IA over the different BCs. Noticeable differences are visible regarding the magnitude, as observed for KE in Fig. 4. The visualization further confirms the difference between BC1 and 3 versus the other conditions, with TAWSS being slightly lower for these two BCs than for the remaining ones.

### Lumen Segmentation

**Fig. 6:**
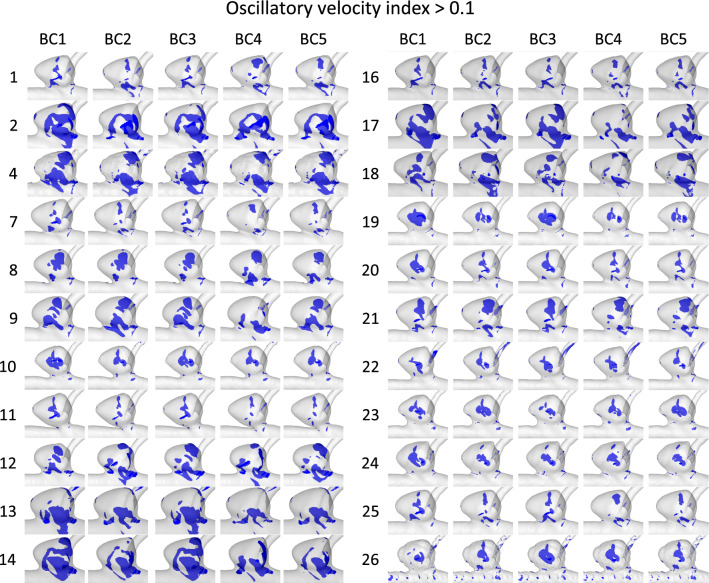
Resultant OVI iso-volume with a threshold of 0.1 for each segmentation of IA E (rows) comparing the five different BCs (columns). In each row the impact of the different BCs on the resulting OVI distribution is shown

To further investigate the influence of the segmentation on the flow parameters and the relevant BCs, OVI iso-volumes (threshold OVI > 0.1) are shown for all outlet BCs and segmentations only of vessel model 3 (see Fig. 6). The distribution of the OVI iso-volumes shows an equal pattern over the segmentations and a high variation of the magnitude. Concerning the impact of the BC, the pattern of the visualized parameter remains equal and only the magnitude slightly differs. BC1 and 3 tend to show larger OVI iso-volumes than the remaining BCs (e.g., groups 9, 17 and 19). Groups 4 and 26 show abnormal behavior which appears to result from non-physiological segmentation. For OVI, it can be observed that different segmentations cause larger deviations than the variation of the BC. Nevertheless, the BC shows an equal impact on each underlying IA and segmentation model.

The distribution of the shown parameters has an equal pattern over the different IAs and segmentations. Their magnitude differs especially for BC1 and 3 compared to the remaining BCs. The latter will be examined further in the subsequent quantitative analysis.

### Interplay Between the Impact of Boundary Condition and Lumen Segmentation

Looking at the qualitative results, an interaction of the two variants (1) BC and (2) lumen segmentation is not recognizable, still an interplay can be found which is analyzed within this section.

**Fig. 7:**
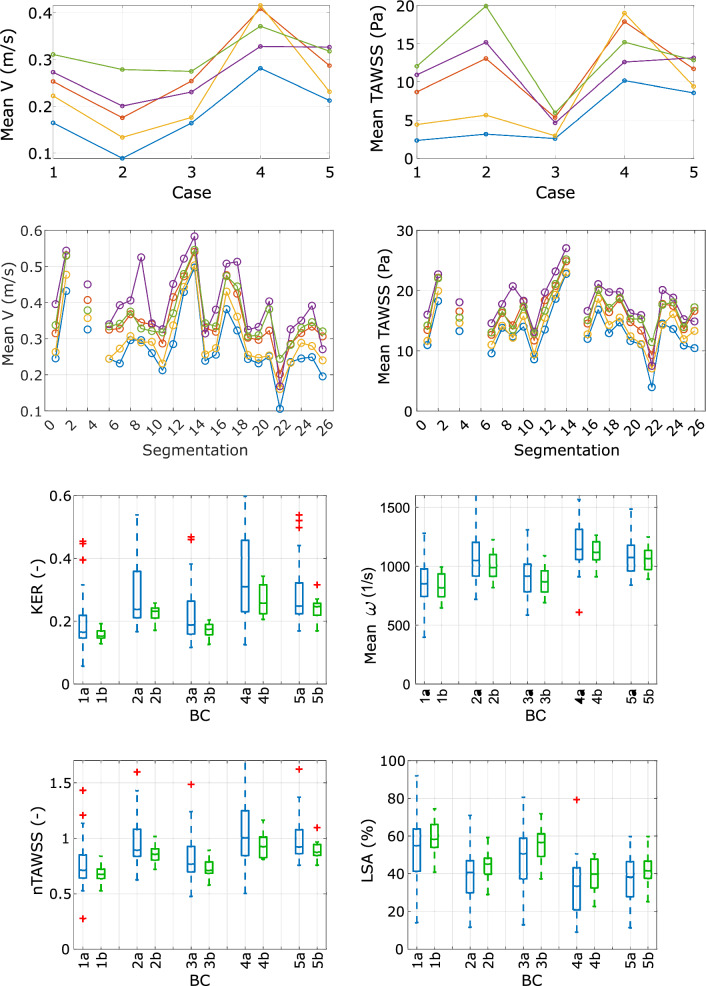
Top: TAWSS and mean V inside the aneurysm for each BC1–5 and IA (first row) and over all segmentations of IA E (second row). Bottom: Boxplots visualizing the impact of the different BCs on the hemodynamic parameters KER, mean ω, normalized TAWSS and LSA within the aneurysm E. Each box contains the results for one BC of all segmentations (a blue bars) and of segmentations where highly deviant results are excluded (b green bars)

First, in Fig. 7a, the two parameters mean V and TAWSS are presented each in a graph over all IAs (first row) and over all segmentations of IA E (second row) for BC1–5. The graphs reveal the differences in magnitude as was already seen in the qualitative analysis ("Outlet Boundary Condition section). BC1 and 3 show lower and BC4 and 5 higher mean V and TAWSS values for the aneurysms A, B, C, E and all segmentations. Only for aneurysm D, BC2 and 3 differ from the other cases with the overall highest value. Graphs for BC2 and 4 of the groups’ segmentation results are close together and in between the remaining BCs (lower than BC5 and higher than BC1 and 3). The most commonly applied zero-pressure BC defines the lower bound over all IAs and segmentations. The advanced flow-splitting based on Chnafa et al. [11] leads to the highest values in these example aneurysms, despite IA D. The graphs show high similarity and differ mostly in a shift along the ordinate. That is, the BC responds similarly to the segmentation, yet a deviation from the overall course can be seen for BC4 and 5.

Segmentation of group 22 reveals the lowest results and appears to show the highest underestimation concerning the relative difference to the reference 2D solution (as mentioned in "Medical Imaging and Segmentation" section: reference figure can be found in [16]). The highest overestimation is shown within the segmentation of group 13. Still, an overestimation over 20% appears for groups 2, 4, 9, 13, 14, 17 and 18, which can be related to higher values of hemodynamic parameters.

Second, in Fig. 7b the impact of the outlet BC on two wall-related and two flow-related hemodynamic parameters inside the aneurysm E for the different segmentations is shown. Boxplots of IA E are presented for all segmentations (a: blue) and for segmentations adjusted for outliers (b: green). The latter are defined as parameter values which deviate more than a three scaled absolute median from the median solution. This affects the segmentations of groups 2, 4, 9, 12, 13, 14, 17, 18 and 22, respectively. Furthermore, groups for which the segmentation results did not meet the requirements of an appropriate surface were excluded as well. This applies for groups 4 and 26, where clear step patterns are present, which affect wall parameters in a non-physiological way. The resulting outliers can be identified with an over- or underestimation when compared to the 2D reference image as presented in [16].

A clear trend in the outlet BCs is visible over the segmentations and the four hemodynamic parameters. BC2, 4, and 5 consistently differ from BC1 and 3, as already seen in the qualitative analysis as well as in Fig. 7a. Mean ω, KER and nAWSS are higher for BC2, 4, and 5, while LSA is lower for BC2, 4 and 5, respectively. BC1 shows the lowest values compared to the remaining BCs for mean ω, KER and nAWSS. Regarding LSA, BC1 shows the highest values.

With a high-fidelity segmentation, namely exclusion of the outliers, the results are more consistent and present a lower scatter. Especially between the different BCs, resulting mean values are less scattered. This trend also strengthens the finding, that each BC responds to each segmentation similarly.

Table 2 lists in the first part the standard deviation of the four hemodynamic parameters from their mean, averaged over all BCs before and after exclusion of BC1 and 3 together with the according reduction (presented in brackets). Three of the parameters are reduced by more than 44%, only mean ω is reduced slightly less.

**Table 2:** Effect of the selected outlet BC (second to third column) or precise segmentation (fourth to last column) on the relative deviation regarding four chosen hemodynamic parameters

Averaged deviation from mean value before and after exclusion of BC1 and 3 (%)				
	nAWSS	LSA	KER	Mean ω
	Before/after (reduction)			
	12.2/3.8 (−69)	11.1/6.3 (−44)	16.2/7.1 (−56)	17.2/13.5 (−22)

Averaged deviation from mean value before and after exclusion of segmentation outliers (%)				
	nAWSS	LSA	KER	Mean ω
	Before/after (reduction)			
BC1	31.4/10.8 (−66)	35.0/14.3 (−59)	51.0/11.5 (−78)	21.8/14.5 (−33)
BC2	23.9/8.7 (−64)	36.9/16.5 (−55)	38.7/10.6 (−73)	19.2/12.9 (−33)
BC3	28.6/12.7 (−56)	37.1/17.2 (−54)	47.4/14.1 (−70)	19.4/13.4 (−31)
BC4	26.2/11.4 (−57)	45.0/20.8 (−54)	40.6/17.6 (−57)	17.6/8.5 (−52)
BC5	21.8/10.1 (−53)	33.8/19.4 (−43)	35.7/14.4 (−60)	15.5/10.2 (−34)

In the second part, the standard deviations from mean value of IA E and all segmentations before and after exclusion of outliers are shown, separated by BC. The according reduction is presented in brackets. A reduction of more than 43% can be seen for all parameters, despite for ω, which is reduced slightly less for BC2, 4, and 5. The results show a consistent range of deviation, independent of the chosen BC. Thus, high-fidelity segmentations lead to a crucial reduction of the parameter deviations. However, deviations of around 10% or sometimes 20% remain, regardless of the selected BC.

## Discussion

Image-based blood flow simulations are increasingly performed to assess apparently patient-specific hemodynamics in neurovascular diseases without harming the individual. Although the underlying techniques provide superior spatial and temporal resolutions compared to state-of-the-art in vivo imaging modalities, divergent findings with respect to the underlying phenomena are reported [1, 27]. Indeed, with the increasing size of the vascular domain considered, the importance of appropriate BCs rises. In most studies claiming to analyze patient-specific neurovascular diseases, only the vessel lumen relates to the actual patient data after being captured using in vivo image acquisition. However, important model assumptions influencing the simulation accuracy (e.g., in- and outflow BCs and wall constitution) are either taken from the literature or acquired from healthy representative volunteers [32]. On top of that, image processing is not overall as realistic as it should be for investigating patient-specific vessel models.

To overcome the limitation of uncertainty in blood flow simulation results, this study focuses on the evaluation of existing outflow BC approaches and real-life image processing results. Specifically, the influence of five outlet methods on the relevant flow and shear parameters associated with IA rupture was assessed. In addition, the contributions of a recent multiple aneurysms challenge [13] were used to demonstrate the potential interaction of the underlying segmentation technique. The analysis of the resulting 120 time-dependent hemodynamic simulations confirms the already reported importance of an appropriate outflow BC and segmentation technique. Variation in the resulting hemodynamic parameters can be associated with over- and under-segmentation, which was previously quantified based on 2D reference images [16].

Despite qualitative similarities with respect to KE or WSS, non-negligible differences become visible due to different outlet BCs. Regardless of the specific IA, the BCs affect the hemodynamics similarly. The patient-specific shape has a clear impact on the individual flow behavior of each IA, but the changes in magnitude still remain similar for each IA (Figs. 4, 5). These findings are supported by a subsequent quantification of two representative hemodynamic parameters (V and TAWSS) for all IAs which also identifies the potential parameter space (recall Fig. 7a). Higher mean ω and KER were obtained when a realistic splitting (BC2, 4 and 5) was applied. Considerably lower values were present for BC1. This goes in line with Saalfeld et al., who developed and investigated advanced splitting techniques [12]. They found out that avoiding the commonly applied zero-pressure assumption and using advanced flow-splitting techniques instead can result in more accurate parameter calculations [11, 12]. Furthermore, the results within this study show that the flow-splitting method based on Murray’s law with an exponent of n = 2 (BC2) can lead to the stronger similarity with the advanced techniques (BC4 and 5).

This result is also consistent when different segmentations of one IA are considered (Figs. 6, 7). The upper and lower quartiles of nAWSS and LSA exhibit a large spreading. This is mainly an effect of poor segmentations which result in outliers and exposes the need for high-fidelity segmentations. Hence, it confirms the primary effect of the segmentation on both the flow and the shear distribution highlighted by Voß et al. [14] and Goubergrits et al. [15] (recall the row-wise comparison in Figs. 6 and 7).

Thus, the standalone impact of BC and segmentation is shown and related to previous studies. Nevertheless, in this study, both effects (outlet BC and segmentation) are assessed to identify their interplay and the potentially dominant one. As presented in Table 2, abandoning unsuitable outlet BCs (1 and 3) reduces deviation substantially for nAWSS (69%) and KER (56%) and slightly less for LSA (−44%) and mean ω (−22%). Omitting low-fidelity segmentations reduces the deviation for nAWSS by 53–66%, for LSA by 54–59% (despite BC5), and for KER by 57–78%. Mean ω is reduced slightly less again. Thus, the presence of poor segmentation causes the influence of BC to be larger. This leads to the assumption that segmentation fidelity has a higher influence than outlet BC choice. Lumen segmentation is therefore highly important to be as realistic as possible, so that the chosen BC has less impact on the resulting hemodynamics.

Consideration of high-fidelity segmentation is particularly important when investigating the effects of geometric markers on rupture, since the analysis of one IA geometry is biased by the underlying segmentation [9]. In particular, the large variations in TAWSS are critical, as this quantity is associated with aneurysm rupture and important for the rupture risk assessment (e.g., Xu et al. [8]). Therefore, dynamic flow effects or even internal fluctuations, which were correlated with increasing rupture risk, might be overlooked. Consequently, previous studies correlating low TAWSS and nAWSS as well as increased LSA with pathological phenomena in IAs might have resulted in different conclusions if a more realistic outlet BC had been chosen [2, 33–35].

Regarding the limitations of this study, first, only five IAs were taken into account and one was selected to be analyzed further using 22 segmentation results. Since this was part of a well-documented study focusing on segmentation variability, still a real-world variability is considered [13–16]. Second, a ground truth is missing since no precise in vivo measurements of the actual patient-specific flow-splitting were available. Therefore, it is assumed that for now the most realistic models can serve as a reference [11, 12]. Third, the blood flow simulations contain further modeling assumptions apart from the outflow BCs, namely the inlet mass flow rates, which are based on literature, the Newtonian blood modeling and the normal-resolution approach (time step size Δt = 1 ms; mesh base size Δx = 0.07–0.09 mm). However, normal-resolution simulations are conventional approaches to assess hemodynamics in IAs [19]. Normal-resolution was considered as sufficient in this study, due to the focus on mean values. An insight into highly-resolved fluctuations as proposed by Valen-Sendstadt et al. [20, 36] is particularly important for highly time-dependent phenomena. Moreover, all computations are carried out under identical settings (except for the outlet BC variation) and fulfill the recommendations for neurovascular hemodynamic simulations recently formulated by Berg et al. [5].

## Conclusion

In this study, the comparison of the hemodynamics based on different segmentation accuracies of the IA lumen, reveals strong variation in hemodynamic patterns. Comparing the impact of five BCs on intracranial hemodynamics reveals little variation in magnitude. When high-fidelity segmentation is performed, the BC has an even lower impact on the parameter deviation. This leads to the conclusion that realistic lumen segmentation has a stronger impact than the chosen outlet BC on hemodynamic predictions. Performing high-fidelity lumen segmentation can be supported by using high-resolved 2D images. Moreover, the application of advanced splitting methods or at least Murray’s law with exponent n = 2 is recommended for neurovascular blood flow simulations.

## References

[1] Etminan N, Rinkel GJ (2016). Unruptured intracranial aneurysms: development, rupture and preventive management. Nat. Rev. Neurol..

[2] Detmer FJ, Chung BJ, Jimenez C, Hamzei-Sichani F, Kallmes D, Putman C, Cebral JR (2019). Associations of hemodynamics, morphology, and patient characteristics with aneurysm rupture stratified by aneurysm location. Neuroradiology.

[3] Liu J, Xiang J, Zhang Y, Wang Y, Li H, Meng H, Yang X (2014). Morphologic and hemodynamic analysis of paraclinoid aneurysms: ruptured versus unruptured. J. Neurointerv. Surg..

[4] Voß S, Glaßer S, Hoffmann T, Beuing O, Weigand S, Jachau K, Preim B, Thévenin D, Janiga G, Berg P (2016). Fluid–structure simulations of a ruptured intracranial aneurysm: constant versus patient-specific wall thickness. Comput. Math. Methods Med..

[5] Berg P, Saalfeld S, Voß S, Beuing O, Janiga G (2019). A review on the reliability of hemodynamic modeling in intracranial aneurysms: why computational fluid dynamics alone cannot solve the equation. Neurosurg. Focus.

[6] Oliveira IL, Santos GB, Gasche JL, Militzer J, Baccin CE (2021). Non-Newtonian blood modeling in intracranial aneurysm hemodynamics: impact on the wall shear stress and oscillatory shear index metrics for ruptured and unruptured cases. J. Biomech. Eng..

[7] Valen-Sendstad K, Piccinelli M, KrishnankuttyRema R, Steinman DA (2015). Estimation of inlet flow rates for image-based aneurysm CFD models: where and how to begin?. Ann. Biomed. Eng..

[8] Xu L, Liang F, Gu L, Liu H (2018). Flow instability detected in ruptured versus unruptured cerebral aneurysms at the internal carotid artery. J. Biomech..

[9] Wan H, Ge L, Huang L, Jiang Y, Leng X, Feng X, Xiang J, Zhang X (2019). Sidewall aneurysm geometry as a predictor of rupture risk due to associated abnormal hemodynamics. Front. Neurol..

[10] Hodis S, Uthamaraj S, Lanzino G, Kallmes DF, Dragomir-Daescu D (2013). Computational fluid dynamics simulation of an anterior communicating artery ruptured during angiography. BMJ Case Rep..

[11] Chnafa C, Brina O, Pereira VM, Steinman DA (2018). Better than nothing: a rational approach for minimizing the impact of outflow strategy on cerebrovascular simulations. Am. J. Neuroradiol..

[12] Saalfeld S, Voß S, Beuing O, Preim B, Berg P (2019). Flow-splitting-based computation of outlet boundary conditions for improved cerebrovascular simulation in multiple intracranial aneurysms. Int. J. Comput. Assist. Radiol. Surg..

[13] Berg P, Voß S, Saalfeld S, Janiga G, Bergersen AW, Valen-Sendstad K, Bruening J, Goubergrits L, Spuler A, Cancelliere NM, Steinman DA, Pereira VM, Chiu TL, Tsang ACO, Chung BJ, Cebral JR, Cito S, Pallarès J, Copelli G, Csippa B, Paál G, Fujimura S, Takao H, Hodis S, Hille G, Karmonik C, Elias S, Kellermann K, Khan MO, Marsden AL, Morales HG, Piskin S, Finol EA, Pravdivtseva M, Rajabzadeh-Oghaz H, Paliwal N, Meng H, Seshadhri S, Howard M, Shojima M, Sugiyama S-I, Niizuma K, Sindeev S, Frolov S, Wagner T, Brawanski A, Qian Y, Wu Y-A, Carlson KD, Dragomir-Daescu D, Beuing O (2018). Multiple Aneurysms AnaTomy CHallenge 2018 (MATCH): Phase I: segmentation. Cardiovasc. Eng. Technol..

[14] Voß S, Beuing O, Janiga G, Berg P (2019). Multiple Aneurysms AnaTomy CHallenge 2018 (MATCH)-Phase Ib: effect of morphology on hemodynamics. PLoS ONE.

[15] Goubergrits L, Hellmeier F, Bruening J, Spuler A, Hege H-C, Voss S, Janiga G, Saalfeld S, Beuing O, Berg P (2019). Multiple Aneurysms AnaTomy CHallenge 2018 (MATCH): uncertainty quantification of geometric rupture risk parameters. BioMed Eng. OnLine.

[16] Berg P, Voß S, Janiga G, Saalfeld S, Bergersen AW, Valen-Sendstad K, Bruening J, Goubergrits L, Spuler A, Chiu TL, Tsang ACO, Copelli G, Csippa B, Paál G, Závodszky G, Detmer FJ, Chung BJ, Cebral JR, Fujimura S, Takao H, Karmonik C, Elias S, Cancelliere NM, Najafi M, Steinman DA, Pereira VM, Piskin S, Finol EA, Pravdivtseva M, Velvaluri P, Rajabzadeh-Oghaz H, Paliwal N, Meng H, Seshadhri S, Venguru S, Shojima M, Sindeev S, Frolov S, Qian Y, Wu Y-A, Carlson KD, Kallmes DF, Dragomir-Daescu D, Beuing O (2019). Multiple Aneurysms AnaTomy CHallenge 2018 (MATCH)-phase II: rupture risk assessment. Int. J. Comput. Assist. Radiol. Surg..

[17] Murray CD (1926). The physiological principle of minimum work: I. The vascular system and the cost of blood volume. Proc. Natl Acad. Sci. USA.

[18] Khan MO, Chnafa C, Gallo D, Molinari F, Morbiducci U, Steinman DA, Valen-Sendstad K (2017). On the quantification and visualization of transient periodic instabilities in pulsatile flows. J. Biomech..

[19] Cebral JR, Mut F, Weir J, Putman CM (2011). Association of hemodynamic characteristics and cerebral aneurysm rupture. Am. J. Neuroradiol..

[20] Valen-Sendstad K, Steinman DA (2014). Mind the gap: impact of computational fluid dynamics solution strategy on prediction of intracranial aneurysm hemodynamics and rupture status indicators. Am. J. Neuroradiol..

[21] Berg, P., C. Roloff, O. Beuing, S. Voss, S.-I. Sugiyama, N. Aristokleous, A. S. Anayiotos, N. Ashton, A. Revell, N. W. Bressloff, A. G. Brown, B. J. Chung, J. R. Cebral, G. Copelli, W. Fu, A. Qiao, A. J. Geers, S. Hodis, D. Dragomir-Daescu, E. Nordahl, Y. Bora Suzen, M. Owais Khan, K. Valen-Sendstad, K. Kono, P. G. Menon, P. G. Albal, O. Mierka, R. Münster, H. G. Morales, O. Bonnefous, J. Osman, L. Goubergrits, J. Pallares, S. Cito, A. Passalacqua, S. Piskin, K. Pekkan, S. Ramalho, N. Marques, S. Sanchi, K. R. Schumacher, J. Sturgeon, H. Švihlová, J. Hron, G. Usera, M. Mendina, J. Xiang, H. Meng, D. A. Steinman, and G. Janiga. The Computational Fluid Dynamics Rupture Challenge 2013—Phase II: variability of hemodynamic simulations in two intracranial aneurysms. *ASME Int. J. Biomech. Eng.* 2015. 10.1115/1.4031794. 10.1115/1.4031794 26473395

[22] Liu H, Lan L, Abrigo J, Ip HL, Soo Y, Zheng D, Wong KS, Wang D, Shi L, Thomas W, Leng X (2021). Comparison of Newtonian and non-Newtonian fluid models in blood flow simulation in patients with intracranial arterial stenosis. Front. Physiol..

[23] Morales HG, Larrabide I, Geers AJ, Aguilar ML, Frangi AF (2013). Newtonian and non-Newtonian blood flow in coiled cerebral aneurysms. J. Biomech..

[24] Byrne G, Mut F, Cebral J (2014). Quantifying the large-scale hemodynamics of intracranial aneurysms. Am. J. Neuroradiol..

[25] Mut F, Löhner R, Chien A, Tateshima S, Viñuela F, Putman C, Cebral J (2011). Computational hemodynamics framework for the analysis of cerebral aneurysms. Int. J. Numer. Methods Biomed. Eng..

[26] Detmer FJ, Chung BJ, Mut F, Slawski M, Hamzei-Sichani F, Putman C, Jiménez C, Cebral JR (2018). Development and internal validation of an aneurysm rupture probability model based on patient characteristics and aneurysm location, morphology, and hemodynamics. Int. J. Comput. Assist. Radiol. Surg..

[27] Cebral JR, Mut F, Weir J, Putman C (2011). Quantitative characterization of the hemodynamic environment in ruptured and unruptured brain aneurysms. Am. J. Neuroradiol..

[28] Sano T, Ishida F, Tsuji M, Furukawa K, Shimosaka S, Suzuki H (2017). Hemodynamic differences between ruptured and unruptured cerebral aneurysms simultaneously existing in the same location: 2 case reports and proposal of a novel parameter oscillatory velocity index. World Neurosurg..

[29] Tanioka S, Ishida F, Kishimoto T, Tsuji M, Tanaka K, Shimosaka S, Toyoda M, Kashiwagi N, Sano T, Suzuki H (2019). Quantification of hemodynamic irregularity using oscillatory velocity index in the associations with the rupture status of cerebral aneurysms. J. Neurointerv. Surg..

[30] Bouillot P, Brina O, Ouared R, Lovblad K-O, Farhat M, Pereira VM (2014). Particle imaging velocimetry evaluation of intracranial stents in sidewall aneurysm: hemodynamic transition related to the stent design. PLoS ONE.

[31] Xiang J, Natarajan SK, Tremmel M, Ma D, Mocco J, Hopkins LN, Siddiqui AH, Levy EI, Meng H (2011). Hemodynamic-morphologic discriminants for intracranial aneurysm rupture. Stroke.

[32] Durka MJ, Wong IH, Kallmes DF, Pasalic D, Mut F, Jagani M, Blanco PJ, Cebral JR, Robertson AM (2018). A data-driven approach for addressing the lack of flow waveform data in studies of cerebral arterial flow in older adults. Physiol. Meas..

[33] Valen-Sendstad K, Mardal K-A, Mortensen M, Reif BAP, Langtangen HP (2011). Direct numerical simulation of transitional flow in a patient-specific intracranial aneurysm. J. Biomech..

[34] Ford MD, Piomelli U (2012). Exploring high frequency temporal fluctuations in the terminal aneurysm of the basilar bifurcation. J. Biomech. Eng..

[35] Poelma C, Watton PN, Ventikos Y (2015). Transitional flow in aneurysms and the computation of haemodynamic parameters. J. R. Soc. Interface.

[36] Valen-Sendstad, K., K.-A. Mardal, and D. A. Steinman. High-resolution CFD detects high-frequency velocity fluctuations in bifurcation, but not sidewall, aneurysms. 2013. 10.1016/j.jbiomech.2012.10.042. 10.1016/j.jbiomech.2012.10.042 23174422

